# The Effects of Dietary Macronutrient Balance on Skin Structure in Aging Male and Female Mice

**DOI:** 10.1371/journal.pone.0166175

**Published:** 2016-11-10

**Authors:** Jonathan Hew, Samantha M. Solon-Biet, Aisling C. McMahon, Kari Ruohonen, David Raubenheimer, J. William O. Ballard, David G. Le Couteur, Caroline Nicholls, Zhe Li, Peter K. M. Maitz, Yiwei Wang, Stephen J. Simpson

**Affiliations:** 1 Burns Research and Reconstructive Surgery, ANZAC Research Institute, Concord Hospital, University of Sydney, Sydney, Australia 2139; 2 Ageing and Alzheimers Institute and ANZAC Research Institute, Concord Hospital, University of Sydney, Sydney, Australia 2139; 3 Charles Perkins Centre and School of Life and Environmental Sciences, University of Sydney, Sydney, Australia; 4 EWOS Innovation, Dirdal 4335, Norway; 5 Faculty of Veterinary Science and Charles Perkins Centre, University of Sydney, Sydney, Australia 2006; 6 School of Biotechnology and Biomolecular Sciences, University of New South Wales, Sydney, Australia; 7 Burns Unit, Concord Repatriation General Hospital, Concord, Australia; University of Melbourne, AUSTRALIA

## Abstract

Nutrition influences skin structure; however, a systematic investigation into how energy and macronutrients (protein, carbohydrate and fat) affects the skin has yet to be conducted. We evaluated the associations between macronutrients, energy intake and skin structure in mice fed 25 experimental diets and a control diet for 15 months using the Geometric Framework, a novel method of nutritional analysis. Skin structure was associated with the ratio of dietary macronutrients eaten, not energy intake, and the nature of the effect differed between the sexes. In males, skin structure was primarily associated with protein intake, whereas in females carbohydrate intake was the primary correlate. In both sexes, the dermis and subcutaneous fat thicknesses were inversely proportional. Subcutaneous fat thickness varied positively with fat intake, due to enlarged adipocytes rather than increased adipocyte number. We therefore demonstrated clear interactions between skin structure and macronutrient intakes, with the associations being sex-specific and dependent on dietary macronutrient balance.

## Introduction

The skin, the largest organ in the body [1], has many crucial functions. It acts as a barrier to microorganisms, toxins, ultraviolet radiation; it prevents water and electrolyte loss and is an active organ of excretion, metabolism, sensation, temperature regulation and immunology [2]. Nutrition is historically one of the most important factors associated with skin diseases and wound healing [3, 4]. It is estimated that 20–50% of patients admitted acutely into a hospital setting have a degree of malnutrition [5] with profound effects on skin structure, function and wound healing [6–9].

Three distinct regions characterize the skin: the epidermis, dermis and subcutaneous fat [10]. The outermost layer of the skin, the epidermis, is mainly populated by keratinocytes and critical for barrier function. The underlying dermis, making up the bulk of the skin, consists primarily of collagen fibres synthesized by dermal fibroblasts, providing tensile strength and mechanical resistance to the skin. Finally, the subcutaneous fat, the deepest layer of the skin, is composed of adipocytes. This tissue has a diversity of functions including not only insulation, thermoregulation and energy storage but also immunological, inflammatory and endocrine qualities [11–13]. Evidence from mouse models [14, 15] and humans [6, 12] demonstrate that all three layers may be affected by variations in the intake of macronutrients.

Malnutrition alters skin structure and can manifest physically with depigmentation, reduced number and atrophy of hair follicles, thinning [16] and importantly, delayed and complicated wound healing [7, 9, 17]. Over-nutrition (i.e. obesity) is also associated with a number of dermatological diseases including psoriasis, dermatitis and skin infections [18, 19]. However, despite the widespread global increase in obesity [20], little is known about the relationship between nutrition, adiposity and skin.

Mice are commonly used as a biological model to investigate skin pathology, due to the difficulties of conducting detailed experimental work in humans. Previous mouse studies investigating nutrition and skin structure have focused mainly on individual macronutrients, particularly high-fat diets [21, 22], or the effect of malnutrition [14, 15]. High fat diets are associated with thinning of the dermis and a reduction in dermal fibroblast proliferation, elasticity [21] and density of type 1 tropocollagen and hyaluronan in the skin [22]. Studies of malnutrition show that both energy deficiency and protein-energy deficiency cause a wasting of the dermis with reductions in collagen density and organization [14, 15].

A large limitation of previous studies has been the manipulation of single nutritional components (i.e. fats or protein) in an attempt to understand an overall response in skin structure. This has in part, been due to a lack of a nutritional framework capable of modeling and analyzing the interactive effects of macronutrients and energy simultaneously. An approach which encompasses the interactive, rather than singular effects of macronutrients, provides a unique insight, and has recently improved our understanding of longevity, reproduction and immunity [23–25]. Here, we use the Geometric Framework for nutrition (GF) to investigate how energy and the dietary balance of protein, carbohydrate and fat affect skin structure in male and female mice. The GF maps nutrition in an n-dimensional space, within which the n components of a diet are represented by separate axes. Phenotypic responses of individuals such as body composition or skin thickness can be mapped as response surfaces onto experimentally derived nutrient intake arrays within this nutrient space. These methods are documented in detail in (Simpson and Le Couteur 2015) [26] and (Simpson and Raubenheimer 2012) [27] and have recently been employed in mice [23–25]. We use the GF to present the first systematic investigation into the individual and interactive effects of total energy, protein, carbohydrate and fat intake on skin structure. This relationship is essential if we are to optimise nutritional interventions to treat dermatological diseases, improve skin quality in the elderly and accelerate wound healing.

## Results

We first set out to characterise the basal state of the skin in response to nutrition, using the GF. A three-dimensional nutrient space (protein, carbohydrate and fat intake) was used to model response surfaces generated by general additive statistical models (GAMs) as described in detail previously in Solon-Biet, McMahon [25] To assist in interpretation, response surfaces are shown as 2D heat maps that cut through the median of the third nutrient axis. Red areas indicate highest values for that response variable, and fall as the colours shift to blue. Isolines in response surfaces indicate areas of equality for the response variable. For example, in surfaces showing dermis thickness, the number shown on each isoline indicates the exact value of the measured variable. If isolines are vertical, the response variable is strongly influenced by the macronutrient of the x axis; likewise if isolines are horizontal, the response variable is strongly influenced by the macronutrient of the y axis. If isolines are at 45 degrees then this indicates that the response variable is largely affected by energy intake (i.e. the sum of energy of the x and y axes). In some response surfaces, we have superimposed nutritional rails that radiate from the origin. These nutritional rails indicate a composition of dietary protein:carbohydrate:fat in which an animal can move along by changing food intake, but cannot alter the ratio of macronutrients ingested. The superimposed nutritional rails represent a specific macronutrient ratio and highlight the relationship between dietary intake and various indicators of skin structure.

### The relationship between dietary macronutrients and skin structure

**Fig 1:**
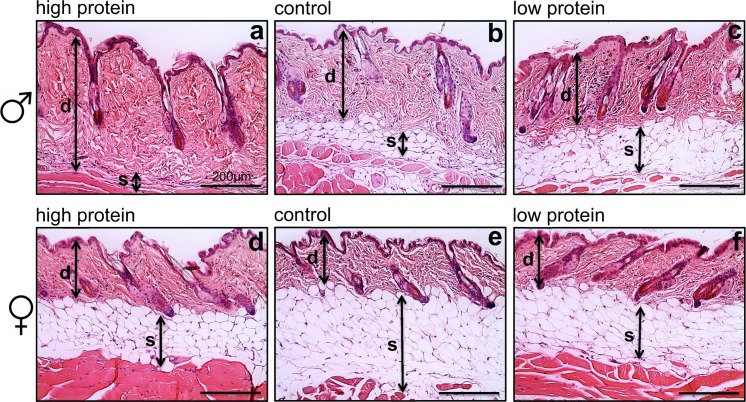
The association between protein intake and male and female skin structure. H&E staining for male mouse skin layers (a-c) and female mouse skin layers (d-f), x20 magnification, scale bar = 200 μm, ‘d’ indicates area of dermis and ‘s’ indicates area of subcutaneous fat. High protein intake significantly increases male dermis thickness and thins the subcutaneous fat. In females, no effect of protein intake on skin structure was identified. Dietary composition of standard chow is protein (21%), carbohydrate (63%) and fat (16%). Mean skin thickness (a) d = 391 μm, s = 54 μm, (b) d = 275 μm s = 90 μm, (c) d = 228 μm, s = 171 μm, (d) d = 203 μm, s = 148 μm, (e) d = 127 μm, s = 233 μm, (f) d = 194 μm, s = 173 μm.

Chow-fed animals showed sex-specific changes in skin structure with males having a significantly thicker dermis (~356 μm) compared to female mice (~168 μm) (P = 0.009, Fig 1B and 1E respectively). The thickness of the subcutaneous fat layer in female mice (~177 μm) and the average area of adipocytes (~767 μm^2^) were also found to be larger than in male mice with subcutaneous fat (~120 μm) and average adipocyte size (~544 μm^2^) although this difference was not significant (P = 0.124 and P = 0.069 respectively).

**Fig 2:**
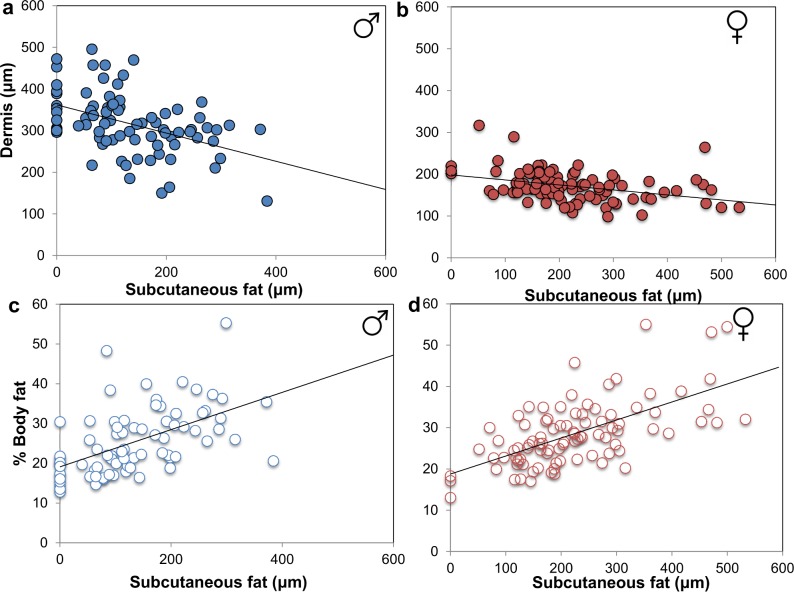
Dermis thickness (μm) and subcutaneous fat thickness (μm) are inversely proportional and correlate with body fat%. Dermis thickness increases with a thinner subcutaneous fat in both (a) males (R^2^ = -0.448; P<0.001) then (b) females (R^2^ = -0.362; P<0.001). Subcutaneous fat increases with increasing body fat % in (c) male and (d) female mice (R^2^ = 0.549; P<0.001 and R^2^ = 0.626; P<0.001, respectively).

To determine the effect of diet on skin structure, we analyzed the thickness of the epidermis, dermis and subcutaneous fat in mice fed experimental diets. We found that macronutrient intake influenced skin structure and that these effects were sex-specific. In males, elevated protein intake was associated with increased epidermis and dermis thickness (P = 0.006 and P<0.001, respectively; Fig 1A–1C; S2 Table). In contrast, the dermis of females were most notably associated with carbohydrate intake (P = 0.044) (Fig 1D–1F; S3 Table). Subcutaneous fat in both sexes were most strongly associated with carbohydrate and fat intake (S2 and S3 Tables) and showed an inverse relationship between subcutaneous fat and dermis thickness (Fig 2A and 2B). Subcutaneous fat deposition was also strongly associated with whole body adiposity (Fig 2C and 2D).

**Fig 3:**
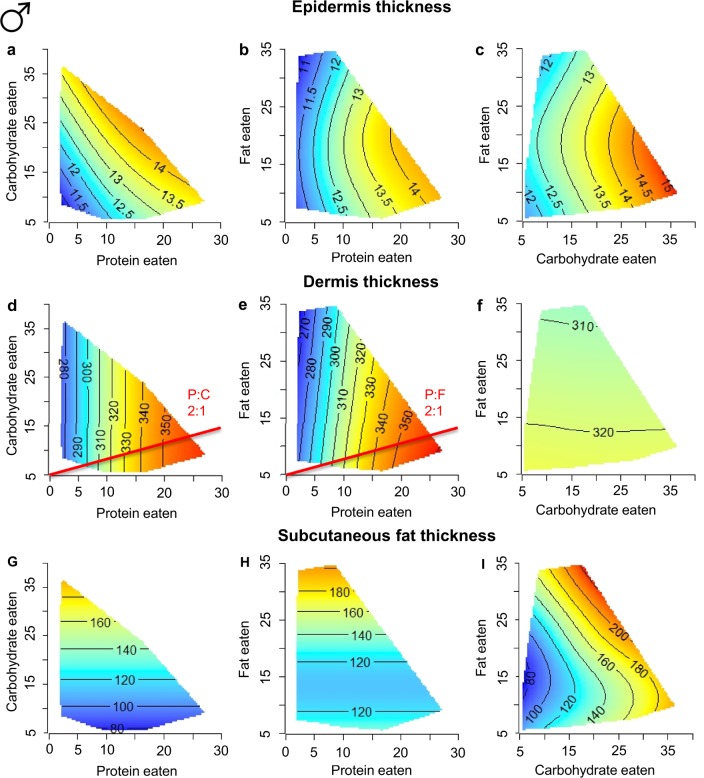
Macronutrients and male skin structure. **Response surfaces showing the association between macronutrient intake (protein, carbohydrate and fat in kJ/d) and male skin structure.** (a-c) epidermis thickness (μm) increases with high protein or carbohydrate intake, (d-f) dermis thickness (μm) increases with high protein intake, (g-i) subcutaneous fat thickness (μm) increases with high carbohydrate or high fat intake. For each 2D slice, the third factor is at its median. Response surfaces are similar to heat maps, red indicates maximum values, blue indicates minimum values. The black lines are isolines, like contour lines on a topographical map, and indicate areas of equality for the response variable e.g. in (a) isolines join areas of equal epidermis thickness. The red lines are nutritional rails and indicate the ratio of macronutrients that maximizes each response. (See also S2 Table).

**Fig 4:**
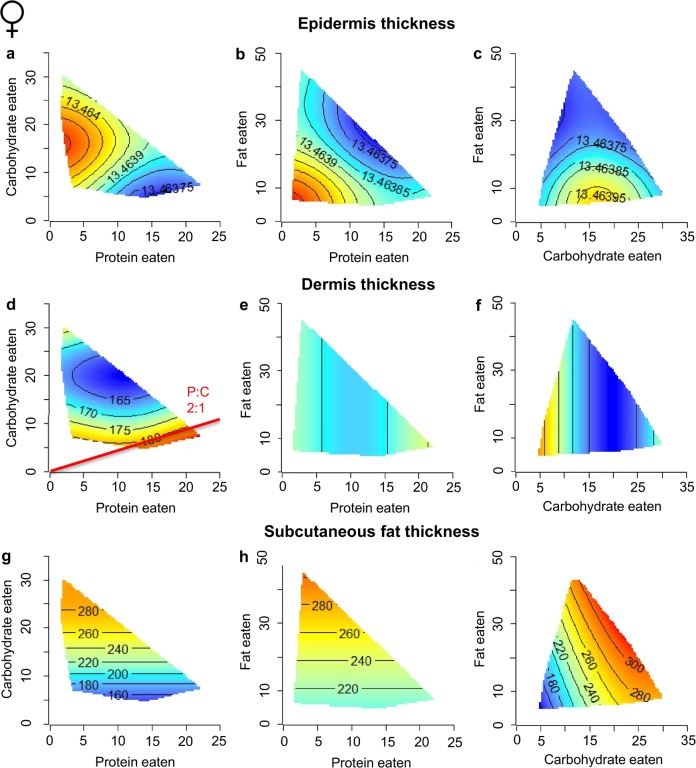
Macronutrients and female skin structure. **Response surfaces showing the association between macronutrient intakes (protein, carbohydrate and fat in kJ/d) and female skin structure.** (a-c) epidermis thickness (μm) shows no variation with macronutrient intake. (d-f) dermis thickness (μm) increases with low carbohydrate intake. (g-i) subcutaneous fat thickness (μm) increases with high carbohydrate or high fat intake. For each 2D slice, the third factor is at its median. Red indicates maximum values, blue indicates minimum values. The red lines indicate the ratio of macronutrients that maximized each response (See also S3 Table).

To determine the contributions of macronutrients and energy to the various skin components, response surfaces were plotted. The effect of macronutrients on the epidermis, dermis and subcutaneous fat layer varied largely between sex (Figs 3 and 4) and were not associated with energy intake but rather specific macronutrients.

In males, epidermis thickness was maximal under high intakes of both protein and carbohydrate (red regions in Fig 3A–3C). Dermis thickness, by contrast, was associated only with protein intake (Fig 3D and 3E). Dermis thickness was thinnest when protein intake was reduced to 5 kJ/d (indicated by a blue colour shift on the surfaces) and maximised when mice consumed a diet with a protein to carbohydrate ratio (P:C) of 2:1. Subcutaneous fat was not associated with protein intake; instead, increased intake of both fat and carbohydrate corresponded with the greatest subcutaneous fat thickness (Fig 3G–3I).

Skin structure in females showed largely opposing responses to those seen in males, with epidermis thickness displaying little variation when compared to males (Fig 4A–4C; S3 Table). Dermis thickness was driven not by protein intake as in males, but solely by carbohydrate intake (P = 0.044; Fig 4D). Maximal dermis thickness occurred when mice consumed a diet with a P:C ratio of 2:1. Subcutaneous fat thickness was greater in females than males across the macronutrient spectrum and similarly dependent on both carbohydrate and fat intake (Fig 4G–4I).

### Dietary macronutrients influence size and number of adipocytes in subcutaneous fat

**Fig 5:**
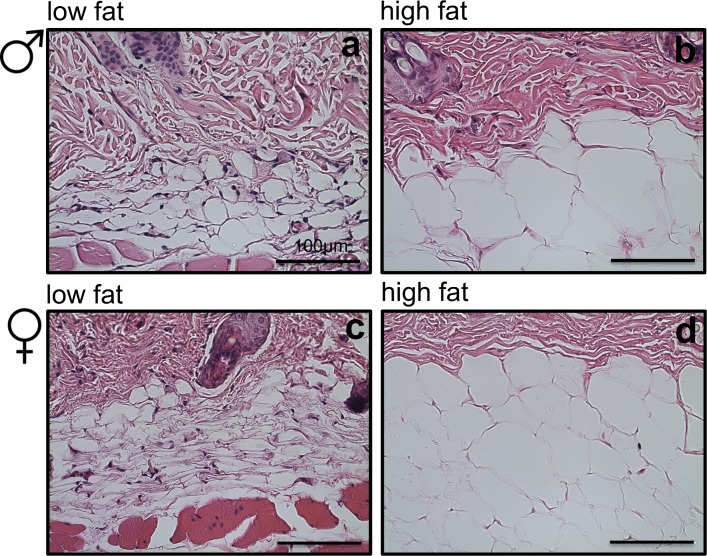
Subcutaneous adipocytes. Representative H&E sections of mouse subcutaneous adipocytes at 40x magnification showing (a) small male adipocytes become greatly engorged with a high fat intake (b). Small female adipocyte (c) become engorged (d) but to a lesser extent than male adipocytes with a high fat diet. scale bar = 100 μm (See also S4 Table).

Given the inverse relationship between dermis thickness and subcutaneous fat, we then measured the size and number of adipocytes in the subcutaneous fat to correlate adipocytes characteristics with the observed changes in dermis structure. Hematoxylin and eosin (H&E) analysis showed that in both male (Fig 5A and 5B) and female mice (Fig 5C and 5D), dietary macronutrients influenced subcutaneous adipocyte size and number.

**Fig 6:**
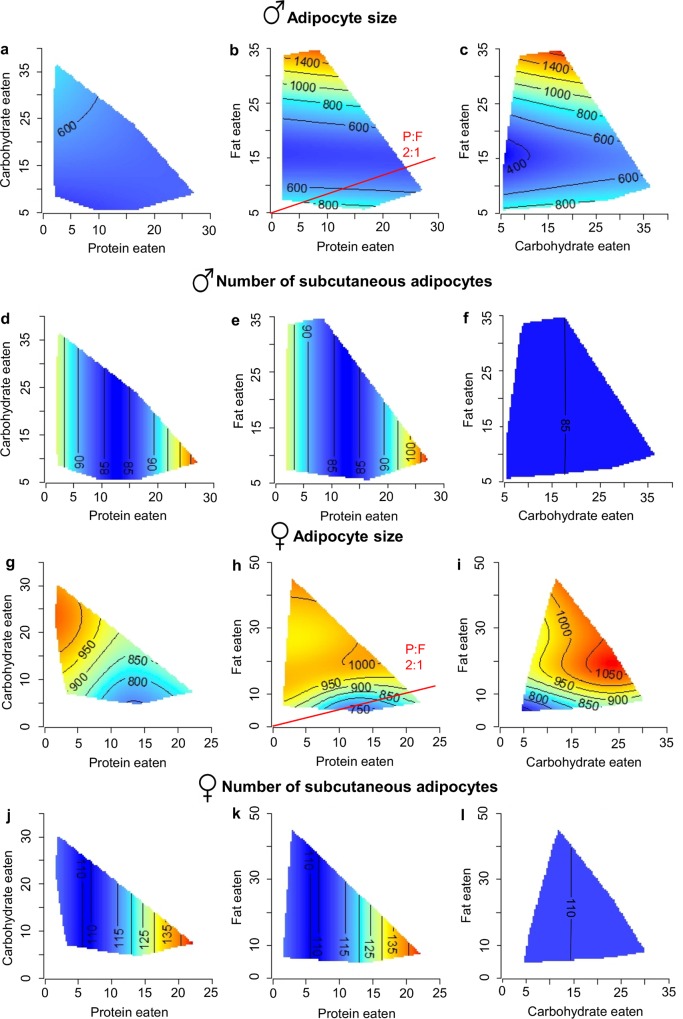
Macronutrient intake influences subcutaneous fat. Response surfaces showing the association of macronutrient intake (protein, carbohydrate and fat in kJ/d) on subcutaneous adipocyte size (μm^2^) and adipocyte numbers (cells/10^5^μm^2^). (a-c) male adipocytes become grossly enlarged with high fat intake whist adipocytes proliferate with high protein intake (d-f; cells/10^5^μm^2^). (g-i) female adipocytes enlarge to a lesser extent than male adipocytes with high carbohydrate or fat intake and proliferate with increasing protein intake (j-i). For each 2D slice, the third factor is at its median. The red line indicates the ratio of macronutrients that minimizes each response. (see also S4 Table)

In both male and female mice, adipocyte size was influenced primarily by fat intake, as indicated by the horizontal inclination of the isolines. Here, adipocytes became engorged as fat intake increased (Fig 6C and 6I; S4 Table). In females, high carbohydrate intake was also associated with increased adipocyte size (Fig 6G and 6I). Adipocyte size in females showed a similar pattern with smaller adipocytes occurring on low fat, medium protein intakes. In both sexes, smallest adipocytes were found when mice consumed a protein to fat ratio (P:F) of 2:1 (Fig 6B and 6H). Although subcutaneous fat thickness was greater in females than males, the maximum adipocyte size was noticeably smaller in females when consuming diets high in fat (Fig 6H and 6B).

When male adipocytes were maximally enlarged, the dermis was at its thinnest and the subcutaneous fat at its thickest (S1 Fig) linking adipocyte enlargement, but not adipocyte number, to the thickening of subcutaneous fat and thinning of the dermis. Similar effects occur in females, with larger adipocytes, but not overall increased adipocyte number, associated with a thinner dermis and thicker subcutaneous fat layer (S1 Fig).

Surprisingly, increased fat intake has no effect on adipocyte numbers in males (Fig 6D–6F) and females (Fig 6J and 6I). Adipocyte numbers were increased under a high protein intake in both males and females, with females having a greater number of adipocytes at all equal macronutrient values, perhaps accounting for a similarly increased thickness of the subcutaneous fat.

## Discussion

Here, we show that the structural changes of the skin are macronutrient dependent and sex specific. Our results demonstrate the applicability of using the GF in the analysis of skin variables and show that skin structure is associated primarily with macronutrient intake, not total energy consumed.

Previous studies have involved a more restricted number of dietary treatments than in the present study, varying either in calories or in a single macronutrient [14, 15, 22, 28, 29]. Furthermore, a comparison of the effect of variations in macronutrient intake on skin structure between sexes has not been attempted previously. In this study, we have used the GF to investigate the interactive effects of macronutrient balance and energy intake on skin structure in male and female mice, and show that skin structure changed markedly with variations in dietary macronutrient intake.

Skin structure and function in humans and mice are similar [30] and are known to differ with sex [31–34]. Such a sex difference was apparent in the present study even in mice fed standard laboratory chow, with male mice having a thicker dermis and females a thicker subcutaneous fat layer with larger adipocytes (see also (30)). We have also shown that male and female skin responds uniquely to macronutrient intake, such that protein intake largely explained skin structural changes in males, whereas carbohydrate intake was significantly associated with skin structure in females.

Sex steroids may underlie the differing responses of male and female skin to variations in dietary intake. We have previously shown that macronutrient balance has substantial effects on steroid production [24]. Using bioassays for estrogenic activity in females and androgenic activity in males, we demonstrated that high protein, high carbohydrate intakes increased steroid-dependent reproductive parameters such as uterine mass in females and seminal vesicle and testes mass in males. In the hair follicle, androgens are aromatized to oestrogen and acting through the oestrogen alpha-receptor increases the proliferative rate of keratinocytes in the basal cell layer of the epidermis [35]. Oestrogens also unfold their actions via regulation of IGF-1 signalling, encouraging fibroblast proliferation [36], enhancing the synthesis of collagen and hyaluronic acid and reducing collagen degradation [37, 38]. Results observed in this present study are consistent with previous findings in that a high protein intake in males increased dermis and epidermis thickness indicating that these mice had higher levels of androgens. In female mice, high carbohydrate intake resulted in a thinner dermis and increased subcutaneous fat. Although this data is consistent with the findings of Azzi in which dermis thickness increased with oophorectomy [33], it is not consistent with literature showing that hypoestrogenism is associated with deterioration of skin condition including thinning of the dermis [37]. Subsequently, alternate mechanisms regulating skin structure must also be affected by nutrient intake.

In addition to systemic hormonal influences, local cytokines released specifically from engorged adipocytes are known to alter skin structure [39]. Adipose tissue can grow through two different mechanisms: adipogenesis or hypertrophy [13]. In this study we show that macronutrient intake may influence which pathway subcutaneous adipose tissue growth occurs and can suggest the impact this has on skin biology. Our results illustrate that a high protein intake encourages adipogenesis and a high fat intake results in adipocyte hypertrophy. In females, a high carbohydrate intake also encouraged adipocyte hypertrophy. The effect this has on skin biology is demonstrated by the correlation between dermis thickness and adipocyte size. As adipocyte size increases dermis thickness decreases. *In vitro* experiments by Ezure *et al* revealed that enlarged adipocytes release free fatty acids, notably palmitic acid which activates Toll-like receptors and alters dermal fibroblast activity [39]. Subsequently, in obese mice with increased subcutaneous tissue there is a reduction in fibroblast proliferation [21]; levels of type 1 tropocollagen and suppression of genes for collagen enzymes and an increase in gene expression for matrix metalloproteinases [22]. Taken together, these processes thin the dermis. In female mice, oestrogen levels increase with high carbohydrate intake; however, adipocytes also enlarge negating the positive oestrogenic effects on the dermis and culminating in a dermis, which is mildly thinned. In contrast, in males, both systemic steroid and local adipocyte proliferation increased with a high protein intake resulting in a notable increase in dermis thickness.

Interestingly, the loss of dermis thickness is a hallmark of intrinsically aging skin [40] and is associated with a functional decline in wound healing, immune response, mechanical strength and elasticity [41]. Azzi *et al* reported in their study that the dermis thickness of young C57BL6 mice was ~ 500 μm for males and ~171 μm for females [33]. Aged control mice in this study had a dermis thickness of ~ 356 μm for males and ~ 168 μm for females showing that the dermis thins with age. In this study, the dermis was thinnest in obese male and female mice with a thickened subcutaneous fat. Wasting of the dermis was minimized in mice consuming a high protein diet where 50% of energy intake was derived from protein and the other 50% from an equal amount of carbohydrate and fat i.e. a ratio of 2:1:1 (P:C:F). This diet was associated with the least dermal attrition, less subcutaneous fat and smaller adipocytes. Further research investigating the clinical utility in the prevention of skin aging or skin rejuvenation is warranted especially considering the increasing prevalence of obesity [20] and ageing of the population [42].

We have shown that macronutrient intake, not total calories, has significant effects on skin structure in both male and female mice using the GF. The response of skin structure to macronutrient intake is sex-specific with protein intake more important in males and carbohydrate intake most significant in females. We present a comprehensive study investigating the interactive effects of energy and macronutrients on skin structure across a wide nutritional spectrum. Upon this foundation, future research exploring various possible endocrine, cellular and molecular mechanisms is required. The advantage of the GF is demonstrated here and will be further utilized for future studies in cutaneous and burn wound healing.

## Materials & Methods

Experimental diets were systematically designed for optimal power in fitting surface response models. Mice were fed one of 25 different diets *ad libitum*, varying in protein (5%-60%, casein and methionine), carbohydrate (16%-75%, sucrose, wheatstarch and dextrinized cornstarch), fat (16%-75%, soya bean oil) content and energy density (S1 Table). Energy density was manipulated by adding indigestible cellulose to yield three different energy regimes (8, 13 and 17 kJ g^-1^).

### Animal model

Mice used in this study were part of a larger cohort of animals that were allowed to age and live on until the end of their natural life to determine lifespan (Solon-Biet et al 2014). 858 male and female C57BL6 mice were obtained from the Animal Resources Centre, WA Australia and housed three per cage in standard approved cages with free access to water, in the Molecular Physiology Unit of the ANZAC Research Institute. The environment was closely controlled at 24–26°C and 44–46% humidity under a 12:12 hr light dark cycle with lights on at 6am. All protocols were approved by the Sydney Local Health District Welfare Committee (Protocol No. 2009/003) and all methods were carried out in accordance with these protocols. Food intake and body weight were measured bi-weekly until 6 months of age and monthly until 15 months of age. To collect food waste for quantification, a custom designed 2-chambre Perspex insert was installed beneath the food hopper of each cage [43]. Over their lifetime mice were *ad libitum-*fed one of 25 diets, checked daily with animals losing more than 20% of their body weight euthanized and the corresponding diet discontinued. These animals and their corresponding diets were not used in further analysis. At 15 months of age, a randomized subset of 178 mice (95 female, 83 male) were anaesthetized using a 1:1 ratio of ketamine and xylazine and euthanized. Animals that failed to thrive were excluded from experiments and analyses. Various tissues including skin samples were collected for histology and analysis using the Geometric Framework. This method of analysis generates statistical power across all dietary groups by plotting individual animals as a single data point on a response surface (Simpson and Raubenheimer 2012).

### Histology

At 15 months of age, mice were anaesthetized and culled. A 1cm^2^ skin biopsy was obtained from the dorsum of each mouse. A commonly used method for processing skin biopsies was employed with samples being fixed in 10% formalin, embedded in paraffin and 5μm sections were stained with hematoxylin and eosin (H&E) [21]. Alignment was carefully adjusted via the angle of sections and appearance of the hair follicles in the dermis, re-embedding and re-sectioning of samples was conducted if it was needed. Sections in which the bulbs of the hair follicles were located below the dermis were excluded from the analysis, controlling for possible effect on thickness of variations in hair cycle staging [44]. Epidermal, dermal and subcutaneous fat thickness was measured using the CAST (Computer Assisted Stereological Toolbox) program V1.10. The meander function was employed to randomize skin cross-sectional areas for measurement. CASTGRID was used to create a random point grid over the skin cross-section and thickness measured at the points overlying the dermis, epidermis or subcutaneous fat perpendicular to the bordering layers. For each layer separately, at least six measurements were obtained for each individual sample [45].

Subcutaneous adipocyte size and number was quantified using EVOS Digital Inverted Fluorescence Microscope (Fisher Scientific). For each section, adipocyte numbers were calculated in 10 randomized fields of view (100x100 μm) at 40x magnification. Fields of view were aligned to start from adipocytes directly under the dermis. The numbers of adipocytes in each field of view were counted. In the event that adipocyte cell membranes were not entirely contained in the field of view, adipocytes whose cell membrane crossed either the deepest boarder from the dermis or the right boarder in the field of view were excluded. The average adipocyte size present in each image was calculated based on the area of the field of view divided by the number of counted cells, and from these figures, the adipocyte surface area per fat sample was calculated based on the average of all 10 images per skin sample.

### Statistical modeling and analysis

First, generalized additive models (GAM) were constructed for epidermis, dermis, subcutaneous fat thickness, adipocyte number and size to check for evidence of different response surfaces for males and females. A full three-way thin-plate spline interaction was fitted with and without sex included, and the two models were compared with a likelihood ratio test. Results indicated substantial differences between males and females in most variables measured (S5 Table). Accordingly, data from males and female have been analysed separately. Response surfaces were generated using non-parametric thin plate splines in R (v3.0.1) and analyzed using General Additive Modeling (S2–S4 Tables) Pearson’s correlations were calculated with SPSS version 21.

## Supporting Information

S1 Fig Related to Fig 6. The relationship between skin structure and subcutaneous adipocyte size and number. In male mice adipocyte size is negatively correlated with dermis thickness (a; P = 0.011) and positively correlated with subcutaneous fat thickness (c; P<0.001). In females, adipocyte size is also greatest when the dermis is the thinnest (e; P = 0.001) and when the subcutaneous fat is the thickest (g; P<0.001). Adipocyte number in females increased with dermis thickness (f; P = 0.004) but not in males (b; P = 0.304). Adipocyte number decreased with increasing subcutaneous fat thickness in females (h; P = 0.016).
(DOCX)

S1 Table Related to experimental procedures. **Experimental diets** showing the % total energy of protein (P), carbohydrate (C) and fat (F). Discontinued diets indicated by -. n = 25/diet.
(DOCX)

S2 Table Related to Fig 2. Coefficients of the GAM associated with male skin thickness.
(DOCX)

S3 Table Related to Fig 3. Coefficients of the GAM associated with female skin thickness.
(DOCX)

S4 Table Related to Fig 4. Coefficients of the GAM associated with male and female adipocytes.
(DOCX)

S5 Table Related to experimental procedures. **Coefficients of the GAM evaluating the effect of sex**. Significant values indicate that the response surfaces in each case differ with sex.
(DOCX)

## References

[1] Gantwerker EA, Hom DB. Skin: histology and physiology of wound healing. Facial Plast Surg Clin North Am. 2011;19(3): 441–453. 10.1016/j.fsc.2011.06.009 21856533

[2] Hwa C, Bauer EA, Cohen DE. Skin biology. Dermatol Ther. 2011;24(5): 464–470. 10.1111/j.1529-8019.2012.01460.x 22353152

[3] Brown KL, Phillips TJ. Nutrition and wound healing. Clin Dermatol. 2010;28(4): 432–439. 10.1016/j.clindermatol.2010.03.028 20620761

[4] Lakdawala N, Babalola O, Fedeles F, McCusker M, Ricketts J, Whitaker-Worth D, et al The role of nutrition in dermatologic diseases: facts and controversies. Clin Dermatol. 2013;31(6): 677–700. 10.1016/j.clindermatol.2013.05.004 24160272

[5] Barker LA, Gout BS, Crowe TC. Hospital malnutrition: prevalence, identification and impact on patients and the healthcare system. Int J Environ Res Public Health. 2011;8(2): 514–527. 10.3390/ijerph8020514 21556200 PMC3084475

[6] Thavaraj V, Sesikeran B. Histopathological changes in skin of children with clinical protein energy malnutrition before and after recovery. J Trop Pediatr. 1989;35(3): 105–108. 2502637 10.1093/tropej/35.3.105

[7] Barbul A, Purtill WA. Nutrition in wound healing. Clin Dermatol. 1994;12(1): 133–140. 8180936 10.1016/0738-081x(94)90264-x

[8] Arnold M, Barbul A. Nutrition and wound healing. Plast Reconstr Surg. 2006;117(7 Suppl): 42S–58S. 10.1097/01.prs.0000225432.17501.6c 16799374

[9] Wild T, Rahbarnia A, Kellner M, Sobotka L, Eberlein T. Basics in nutrition and wound healing. Nutrition. 2010;26(9): 862–866. 10.1016/j.nut.2010.05.008 20692599

[10] Khavkin J, Ellis DA. Aging skin: histology, physiology, and pathology. Facial Plast Surg Clin North Am. 2011;19(2): 229–234. 10.1016/j.fsc.2011.04.003 21763983

[11] Ralf Paus L, Klein J, Permana PA, Owecki M, Chaldakov GN, Böhm M, et al What are subcutaneous adipocytes really good for…? Exp Dermatol. 2007;16(1): 45–47. 10.1111/j.1600-0625.2006.00519_1.x 17181636

[12] Booth A, Magnuson A, Foster M. Detrimental and protective fat: body fat distribution and its relation to metabolic disease. Horm Mol Biol Clin Investig. 2014;17(1): 13–27. 10.1515/hmbci-2014-0009 25372727

[13] Rivera-Gonzalez G, Shook B, Horsley V. Adipocytes in skin health and disease. Cold Spring Harb Perspect Med. 2014;4(3): 1–18. 10.1101/cshperspect.a015271 PMC3935392 24591537

[14] Thomas JR. Effects of age and diet on rat skin histology. Laryngoscope. 2005;115(3): 405–411. 10.1097/01.mlg.0000157845.86154.48 15744148

[15] Leite SN, Jordao Junior AA, Andrade TA, Masson Ddos S, Frade MA. Experimental models of malnutrition and its effect on skin trophism. An Bras Dermatolo 2011;86(4): 681–688. 10.1590/s0365-05962011000400009 21987133

[16] McLaren DS. Skin in protein energy malnutrition. Arch Dermatol. 1987;123(12): 1674–1676a. 3120652

[17] Williams JZ, Barbul A. Nutrition and wound healing. Surg Clin North Am. 2003;83(3): 571–596. 10.1016/S0039-6109(02)00193-7 12822727

[18] Yosipovitch G, DeVore A, Dawn A. Obesity and the skin: skin physiology and skin manifestations of obesity. J Am Acad Dermatol. 2007;56(6): 901–916. 10.1016/j.jaad.2006.12.004 17504714

[19] Scheinfeld NS. Obesity and dermatology. Clin Dermatol. 2004;22(4): 303–309. 10.1016/j.clindermatol.2004.01.001 15475230

[20] Ng M, Fleming T, Robinson M, Thomson B, Graetz N, Margono C, et al Global, regional, and national prevalence of overweight and obesity in children and adults during 1980–2013: a systematic analysis for the Global Burden of Disease Study 2013. Lancet. 2014;384(9945): 766–781. 10.1016/S0140-6736(14)60460-8 24880830 PMC4624264

[21] Ezure T, Amano S. Increased subcutaneous adipose tissue impairs dermal function in diet-induced obese mice. Exp Dermatol. 2010;19(10): 878–882. 10.1111/j.1600-0625.2009.00970.x 19758317

[22] Yamane T, Kobayashi-Hattori K, Oishi Y, Takita T. High-fat diet reduces levels of type I tropocollagen and hyaluronan in rat skin. Mol Nutr Food Res. 2010;54 Suppl 1: S53–S61. 10.1002/mnfr.201000022 20397200

[23] Le Couteur DG, Tay SS, Solon-Biet S, Bertolino P, McMahon AC, Cogger VC, et al The influence of macronutrients on splanchnic and hepatic lymphocytes in aging mice. J Gerontol A Biol Sci Med Sci. 2015;70(12): 1499–1507. 10.1093/gerona/glu196 25335766

[24] Solon-Biet SM, Walters KA, Simanainen UK, McMahon AC, Ruohonen K, Ballard JW, et al Macronutrient balance, reproductive function, and lifespan in aging mice. Proc Natl Acad Sci U S A. 2015;112(11): 3481–3486. 10.1073/pnas.1422041112 25733862 PMC4371964

[25] Solon-Biet SM, McMahon AC, Ballard JW, Ruohonen K, Wu LE, Cogger VC, et al The ratio of macronutrients, not caloric intake, dictates cardiometabolic health, aging, and longevity in ad libitum-fed mice. Cell Metab. 2014;19(3): 418–430. 10.1016/j.cmet.2014.02.009 24606899 PMC5087279

[26] Simpson SJ, Le Couteur DG, Raubenheimer D. Putting the balance back in diet. Cell. 2015;161(1): 18–23. 10.1016/j.cell.2015.02.033 25815981

[27] Simpson SJ, Raubenheimer D. The nature of nutrition A unifying framework from animal adaption to human obesity. Princeton University Press; 2012 p. 248.

[28] Loewenthal LA, Montagna W. Effects of caloric restriction on skin and hair growth in mice. J Invest Dermatol. 1955;24(4): 429–433. 14367935 10.1038/jid.1955.58

[29] Sugiyama A, Fujita Y, Kobayashi T, Ryu M, Suzuki Y, Masuda A, et al Effect of protein malnutrition on the skin epidermis of hairless mice. J Vet Med Sci. 2011;73(6): 831–835. 21289473 10.1292/jvms.10-0399

[30] Dao H Jr., Kazin RA. Gender differences in skin: a review of the literature. Gend Med. 2007;4(4): 308–328. 18215723 10.1016/s1550-8579(07)80061-1

[31] Sjostrom L, Smith U, Krotkiewski M, Bjorntorp P. Cellularity in different regions of adipose tissue in young men and women. Metabolism. 1972;21(12): 1143–1153. 4629846 10.1016/0026-0495(72)90109-6

[32] Shuster S, Black MM, McVitie E. The influence of age and sex on skin thickness, skin collagen and density. Br J Dermatol. 1975;93(6): 639–643. 1220811 10.1111/j.1365-2133.1975.tb05113.x

[33] Azzi L, El-Alfy M, Martel C, Labrie F. Gender differences in mouse skin morphology and specific effects of sex steroids and dehydroepiandrosterone. J Clin Investig Dermatol. 2005;124(1): 22–27. 10.1111/j.0022-202X.2004.23545.x 15654949

[34] Makrantonaki E, Brink TC, Zampeli V, Elewa RM, Mlody B, Hossini AM, et al Identification of biomarkers of human skin ageing in both genders. Wnt signalling—a label of skin ageing? PLoS One. 2012;7(11): e50393 10.1371/journal.pone.0050393 23226273 PMC3511529

[35] Moverare S, Lindberg MK, Faergemann J, Gustafsson JA, Ohlsson C. Estrogen receptor alpha, but not estrogen receptor beta, is involved in the regulation of the hair follicle cycling as well as the thickness of epidermis in male mice. J Invest Dermatol. 2002;119(5): 1053–1058. 10.1046/j.1523-1747.2002.00637.x 12445192

[36] Makrantonaki E, Vogel K, Fimmel S, Oeff M, Seltmann H, Zouboulis CC. Interplay of IGF-I and 17beta-estradiol at age-specific levels in human sebocytes and fibroblasts in vitro. Exp Gerontol. 2008;43(10): 939–946. 10.1016/j.exger.2008.07.005 18755261

[37] Stevenson S, Thornton J. Effect of estrogens on skin aging and the potential role of SERMs. Clin Interv Aging. 2007;2(3): 283–297. 18044179 10.2147/cia.s798 PMC2685269

[38] Shu YY, Maibach H. Estrogens and skin theraputic options Am J Clin Dermatol. 2011;12(5): 297–311. 10.2165/11589180-000000000-00000 21714580

[39] Ezure T, Amano S. Negative regulation of dermal fibroblasts by enlarged adipocytes through release of free fatty acids. J Invest Dermatol. 2011;131: 2004–2009. 10.1038/jid.2011.145 21697886

[40] Robert L, Labat-Robert J, Robert AM. Physiology of skin aging. Pathol Biol (Paris). 2009;57(4): 336–341. 19046830 10.1016/j.patbio.2008.09.007

[41] Farage MA, Miller KW, Elsner P, Maibach HI. Intrinsic and extrinsic factors in skin ageing: a review. Int J Cosmet Sci. 2008;30(2): 87–95. 10.1111/j.1468-2494.2007.00415.x 18377617

[42] Ezeh AC, Bongaarts J, Mberu B. Global population trends and policy options. Lancet. 2012;380(9837): 142–148. 10.1016/S0140-6736(12)60696-5 22784532

[43] Sorensen A, Mayntz D, Raubenheimer D, Simpson SJ. Protein-leverage in mice: the geometry of macronutrient balancing and consequences for fat deposition. Obesity. 2008;16(3): 566–571. 10.1038/oby.2007.58 18239565

[44] Muller-Rover S, Handjiski B, van der Veen C, Eichmuller S, Foitzik K, McKay IA, et al A comprehensive guide for the accurate classification of murine hair follicles in distinct hair cycle stages. J Invest Dermatol. 2001;117(1): 3–15. 10.1046/j.0022-202x.2001.01377.x 11442744

[45] Simanainen U, Ryan T, Li D, Suarez FG, Gao YR, Watson G, et al Androgen receptor actions modify skin structure and chemical carcinogen-induced skin cancer susceptibility in mice. Horm Cancer. 2015;6(1): 45–53. 10.1007/s12672-014-0210-1 25563841 PMC10355911

