# Supplementary material for: The Effects of Dietary Macronutrient Balance on Skin Structure in Aging Male and Female Mice
Source: PLoS One. 2016 Nov 10;11(11):e0166175. doi: 10.1371/journal.pone.0166175 (PMC5104383; doi:10.1371/journal.pone.0166175)
Supplement: S1 Fig — The relationship between skin structure and subcutaneous adipocyte size and number. In male mice adipocyte size is negatively correlated with dermis thickness (a; P = 0.011) and positively correlated with subcutaneous fat thickness (c; P<0.001). In females, adipocyte size is also greatest when the dermis is the thinnest (e; P = 0.001) and when the subcutaneous fat is the thickest (g; P<0.001). Adipocyte number in females increased with dermis thickness (f; P = 0.004) but not in males (b; P = 0.304). Adipocyte number decreased with increasing subcutaneous fat thickness in females (h; P = 0.016). (DOCX) [file pone.0166175.s001.docx]

**
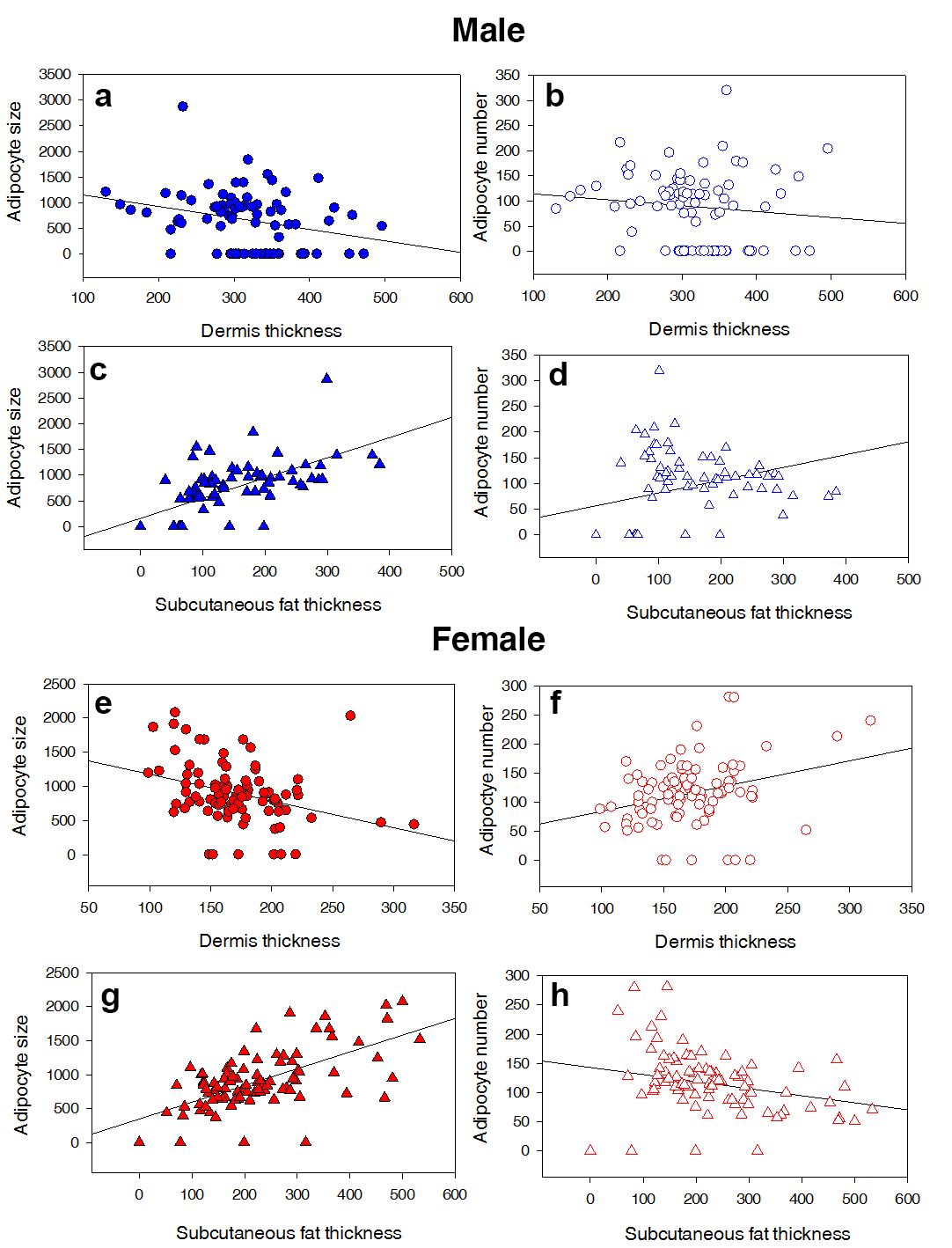
**

**S1 Fig, related to Fig 6. The relationship between skin structure and subcutaneous adipocyte size and number.** In male mice adipocyte size is negatively correlated with dermis thickness (a; P=0.011) and positively correlated with subcutaneous fat thickness (c; P<0.001). In females, adipocyte size is also greatest when the dermis is the thinnest (e; P=0.001) and when the subcutaneous fat is the thickest (g; P<0.001). Adipocyte number in females increased with dermis thickness (f; P=0.004) but not in males (b; P=0.304). Adipocyte number decreased with increasing subcutaneous fat thickness in females (h; P=0.016).
