# Supplementary material for: The Effects of Dietary Macronutrient Balance on Skin Structure in Aging Male and Female Mice
Source: PLoS One. 2016 Nov 10;11(11):e0166175. doi: 10.1371/journal.pone.0166175 (PMC5104383; doi:10.1371/journal.pone.0166175)
Supplement: S1 Table — Experimental diets showing the % total energy of protein (P), carbohydrate (C) and fat (F). Discontinued diets indicated by -. n = 25/diet. (DOCX) [file pone.0166175.s002.docx]

**S1 Table, related to experimental procedures.** Experimental diets showing the % total energy of protein (P), carbohydrate (C) and fat (F). Discontinued diets indicated by -.

| **Macronutrient composition of diets** | | | |
| --- | --- | --- | --- |
| Composition | Low energy (8 kJ/g^-1^) | Medium energy (13 kJ/g^-1^) | High energy (17 kJ/g^-1^) |
| % P/C/F | kJ/g^-1^ P/C/F | kJ/g^-1^ P/C/F | kJ/g^-1^ P/C/F |
| 60/20/20 | 5.0/1.7/1.7 | 7.5/2.5/2.5 | 10.0/3.4/3.4 |
| 42/29/29 | 3.5/2.4/2.4 | 5.3/3.6/3.6 | 7.0/4.9/4.9 |
| 33/47/20 | 2.8/4.0/1.7 | 4.2/6.0/2.51 | 5.5/8.0/3.4 |
| 33/20/47 | 2.8/1.7/4.0 | 4.2/2.5/6.0 | 5.5/3.4/8.0 |
| 14/57/29 | 1.2/4.8/2.4 | 1.8/7.2/3.6 | 2.4/9.5/4.9 |
| 14/29/57 | 1.2/2.4/4,8 | 1.8/3.6/7.2 | 2.4/4.8/9.5 |
| 23/38/38 | 1.9/3.2/3.2 | 2.9/4.8/4.8 | 3.9/6.4/6.4 |
| 5/75/20 | - | 0.6/9.4/2.5 | 0.8/12.6/3.4 |
| 5/20/75 | - | - | 0.8/3.4/12.6 |
| 5/48/48 | - | - | 0.8/8.0/8.0 |
