# Supplementary material for: The Effects of Dietary Macronutrient Balance on Skin Structure in Aging Male and Female Mice
Source: PLoS One. 2016 Nov 10;11(11):e0166175. doi: 10.1371/journal.pone.0166175 (PMC5104383; doi:10.1371/journal.pone.0166175)
Supplement: S2 Table — Coefficients of the GAM associated with male skin thickness. (DOCX) [file pone.0166175.s003.docx]

**S2 Table, related to Fig 2.** Coefficients of the GAM associated with male skin thickness.

| **Male** | | | | |
| --- | --- | --- | --- | --- |
| **Epidermis (μm) vs macronutrient intake** | | | | |
|  | edf | Ref.df | F | p-value |
| s(eaten.P) | 0.98 | 8.00 | 0.74 | 0.0063 |
| s(eaten.C) | 0.83 | 8.00 | 0.70 | 0.0907 |
| s(eaten.F) | 0.00 | 8.00 | 0.00 | 0.5292 |
| s(eaten.P,eaten.C) | 0.00 | 3.00 | 0.00 | 0.3789 |
| s(eaten.P,eaten.F) | 1.00 | 3.00 | 0.88 | 0.1012 |
| s(eaten.C,eaten.F) | 0.00 | 3.00 | 0.00 | 0.3440 |
| s(eaten.P,eaten.C,eaten.F) | 0.00 | 10.00 | 0.00 | 0.9407 |
| **Dermis (μm) vs macronutrient intake** | | | | |
|  | edf | Ref.df | F | p-value |
| s(eaten.P) | 1.46 | 8.00 | 1.34 | 0.0008 |
| s(eaten.C) | 0.00 | 8.00 | 0.00 | 0.5717 |
| s(eaten.F) | 0.40 | 8.00 | 0.09 | 0.1856 |
| s(eaten.P,eaten.C) | 0.05 | 3.00 | 0.05 | 0.2859 |
| s(eaten.P,eaten.F) | 0.00 | 3.00 | 0.00 | 0.6185 |
| s(eaten.C,eaten.F) | 0.00 | 3.00 | 0.00 | 0.9205 |
| s(eaten.P,eaten.C,eaten.F) | 0.00 | 10.00 | 0.00 | 0.4779 |
| **Subcutaneous fat (μm) vs macronutrient intake** | | | | |
|  | edf | Ref.df | F | p-value |
| s(eaten.P) | 0.00 | 8.00 | 0.00 | 0.7857 |
| s(eaten.C) | 0.89 | 8.00 | 1.10 | 0.0022 |
| s(eaten.F) | 1.61 | 8.00 | 0.70 | 0.0283 |
| s(eaten.P,eaten.C) | 0.00 | 3.00 | 0.00 | 0.6764 |
| s(eaten.P,eaten.F) | 0.00 | 3.00 | 0.00 | 0.3773 |
| s(eaten.C,eaten.F) | 0.71 | 3.00 | 0.00 | 0.3584 |
| s(eaten.P,eaten.C,eaten.F) | 0.00 | 10.00 | 0.00 | 0.5004 |
