# Supplementary material for: The Effects of Dietary Macronutrient Balance on Skin Structure in Aging Male and Female Mice
Source: PLoS One. 2016 Nov 10;11(11):e0166175. doi: 10.1371/journal.pone.0166175 (PMC5104383; doi:10.1371/journal.pone.0166175)
Supplement: S3 Table — Coefficients of the GAM associated with female skin thickness. (DOCX) [file pone.0166175.s004.docx]

**S3 Table, related to Fig 3.** Coefficients of the GAM associated with female skin thickness.

| **Female** | | | | |
| --- | --- | --- | --- | --- |
| **Epidermis (μm) vs macronutrient intake** | | | | |
|  | edf | Ref.df | F | p-value |
| s(eaten.P) | 0.00 | 8.00 | 0.00 | 0.5290 |
| s(eaten.C) | 0.00 | 8.00 | 0.00 | 0.6890 |
| s(eaten.F) | 0.00 | 8.00 | 0.00 | 0.9892 |
| s(eaten.P,eaten.C) | 0.00 | 3.00 | 0.00 | 0.4670 |
| s(eaten.P,eaten.F) | 0.00 | 3.00 | 0.00 | 0.8221 |
| s(eaten.C,eaten.F) | 0.00 | 3.00 | 0.00 | 0.4008 |
| s(eaten.P,eaten.C,eaten.F) | 0.00 | 10.00 | 0.00 | 0.8100 |
| **Dermis (μm) vs macronutrient intake** | | | | |
|  | edf | Ref.df | F | p-value |
| s(eaten.P) | 0.00 | 8.00 | 0.00 | 0.3871 |
| s(eaten.C) | 0.89 | 8.00 | 0.23 | 0.0440 |
| s(eaten.F) | 0.00 | 8.00 | 0.00 | 0.4509 |
| s(eaten.P,eaten.C) | 0.94 | 3.00 | 0.47 | 0.1069 |
| s(eaten.P,eaten.F) | 0.00 | 3.00 | 0.00 | 0.4312 |
| s(eaten.C,eaten.F) | 0.00 | 3.00 | 0.00 | 0.4656 |
| s(eaten.P,eaten.C,eaten.F) | 0.00 | 10.00 | 0.00 | 0.5932 |
| **Subcutaneous fat (μm) vs macronutrient intake** | | | | |
|  | edf | Ref.df | F | p-value |
| s(eaten.P) | 0.00 | 8.00 | 0.00 | 0.7410 |
| s(eaten.C) | 1.64 | 8.00 | 2.18 | 0.0001 |
| s(eaten.F) | 0.78 | 8.00 | 0.45 | 0.0338 |
| s(eaten.P,eaten.C) | 0.00 | 3.00 | 0.00 | 0.5611 |
| s(eaten.P,eaten.F) | 0.00 | 3.00 | 0.00 | 0.6192 |
| s(eaten.C,eaten.F) | 0.00 | 3.00 | 0.00 | 0.7339 |
| s(eaten.P,eaten.C,eaten.F) | 0.00 | 10.00 | 0.00 | 0.4472 |
