# Supplementary material for: The Effects of Dietary Macronutrient Balance on Skin Structure in Aging Male and Female Mice
Source: PLoS One. 2016 Nov 10;11(11):e0166175. doi: 10.1371/journal.pone.0166175 (PMC5104383; doi:10.1371/journal.pone.0166175)
Supplement: S4 Table — Coefficients of the GAM associated with male and female adipocytes. (DOCX) [file pone.0166175.s005.docx]

**S4 Table, related to Fig 4.** Coefficients of the GAM associated with male and female adipocytes.

| **Male** | | | | |
| --- | --- | --- | --- | --- |
| **Adipocyte size (μm2) vs macronutrient intake** | | | | |
|  | edf | Ref.df | F | p-value |
| s(eaten.P) | 0.00 | 8.00 | 0.00 | 1.0000 |
| s(eaten.C) | 0.42 | 8.00 | 0.42 | 0.0480 |
| s(eaten.F) | 2.39 | 8.00 | 2.64 | 0.0000 |
| s(eaten.P,eaten.C) | 0.00 | 3.00 | 0.00 | 0.4903 |
| s(eaten.P,eaten.F) | 0.00 | 3.00 | 0.00 | 0.4574 |
| s(eaten.C,eaten.F) | 0.00 | 3.00 | 0.00 | 0.6696 |
| s(eaten.P,eaten.C,eaten.F) | 1.54 | 10.00 | 0.00 | 0.3506 |
| **Number of subcutaneous adipocytes (cells/10^5 μm2) vs macronutrient intake** | | | | |
|  | edf | Ref.df | F | p-value |
| s(eaten.P) | 3.59 | 8.00 | 0.20 | 0.1910 |
| s(eaten.C) | 0.00 | 8.00 | 0.00 | 1.0000 |
| s(eaten.F) | 0.01 | 8.00 | 0.00 | 0.7280 |
| s(eaten.P,eaten.C) | 0.00 | 3.00 | 0.00 | 0.4680 |
| s(eaten.P,eaten.F) | 0.00 | 3.00 | 0.00 | 0.6990 |
| s(eaten.C,eaten.F) | 0.00 | 3.00 | 0.00 | 0.8400 |
| s(eaten.P,eaten.C,eaten.F) | 0.00 | 10.00 | 0.00 | 0.5550 |
| **Female** | | | | |
| **Adipocyte size (μm2) vs macronutrient intake** | | | | |
|  | edf | Ref.df | F | p-value |
| s(eaten.P) | 0.00 | 8.00 | 0.00 | 0.9493 |
| s(eaten.C) | 0.92 | 8.00 | 0.28 | 0.0859 |
| s(eaten.F) | 0.52 | 8.00 | 0.13 | 0.0986 |
| s(eaten.P,eaten.C) | 0.00 | 3.00 | 0.00 | 0.4605 |
| s(eaten.P,eaten.F) | 1.28 | 3.00 | 1.09 | 0.0536 |
| s(eaten.C,eaten.F) | 0.00 | 3.00 | 0.00 | 0.7541 |
| s(eaten.P,eaten.C,eaten.F) | 0.00 | 10.00 | 0.00 | 0.8737 |
| **Number of subcutaneous adipocytes (cells/10^5 μm2) vs macronutrient intake** | | | | |
|  | edf | Ref.df | F | p-value |
| s(eaten.P) | 1.25 | 8.00 | 0.41 | 0.0739 |
| s(eaten.C) | 0.00 | 8.00 | 0.00 | 0.3316 |
| s(eaten.F) | 0.00 | 8.00 | 0.00 | 0.7936 |
| s(eaten.P,eaten.C) | 0.00 | 3.00 | 0.00 | 0.4247 |
| s(eaten.P,eaten.F) | 0.00 | 3.00 | 0.00 | 0.7382 |
| s(eaten.C,eaten.F) | 0.00 | 3.00 | 0.00 | 0.7660 |
| s(eaten.P,eaten.C,eaten.F) | 0.00 | 10.00 | 0.00 | 0.6119 |
