# Supplementary material for: The Effects of Dietary Macronutrient Balance on Skin Structure in Aging Male and Female Mice
Source: PLoS One. 2016 Nov 10;11(11):e0166175. doi: 10.1371/journal.pone.0166175 (PMC5104383; doi:10.1371/journal.pone.0166175)
Supplement: S5 Table — Coefficients of the GAM evaluating the effect of sex. Significant values indicate that the response surfaces in each case differ with sex. (DOCX) [file pone.0166175.s006.docx]

**S5 Table, related to experimental procedures.** Coefficients of the GAM evaluating the effect of sex. Significant values indicate that the response surfaces in each case differ with sex.

| **Variable** | **Resid DF** | **Resid Dev** | **Df Deviance** | **Pr(>Chi)** |
| --- | --- | --- | --- | --- |
| Epidermis | 28.41 | 55.13 | 13.84 | 0.0000 |
| Dermis | 8.52 | 771.00 | 20.89 | 0.0000 |
| Subcutaneous fat | 47.28 | 269182.00 | 0.91 | 0.1217 |
| Adipocyte size | 20.85 | 354000.00 | 27.84 | 0.0000 |
| Adipocyte number | 45.43 | 152413.00 | 0.88 | 0.2054 |
